# Prevalence of tooth erosion and associated factors in 13-16-year old adolescents in Greece

**DOI:** 10.4317/jced.50802

**Published:** 2012-07-01

**Authors:** Nikolaos A. Chrysanthakopoulos

**Affiliations:** 1 Dental Surgeon DDSc, Resident in Maxillofacial and Oral Surgery, 401 General Military Hospital of Athens, Athens.

## Abstract

Objectives: The aim of this study was to estimate the prevalence of dental erosion and to investigate possible associations among dental erosion and medical history, dietary and lifestyle habits in a sample of adolescents in Greece. Study design: The study sample consisted of 770 adolescents, 374 boys and 396 girls aged 13 to 16 years. All individuals were clinically examined and answered questions regarding their medical history, rate and frequency of drinks and food consumption and lifestyle habits. Statistical analysis of the questionnaire items was performed by using the multiple logistic regression analysis model. Results: Two hundreds and sixty adolescents were diagnosed as having dental erosion, giving a prevalence rate 33.8%. The habit of holding drinks in the mouth before swallowing [OR=2.85, 95% CI=1.45-5.58] (p=0.002), the ingestion of acidic drinks at bedtime [OR=0.24, 95% CI=0.11-0.53] (p=0.000), the consumption of car- bonated drinks [OR=3.99, 95% CI=1.37-11.59] (p=0.011) and fruit juices [OR=0.12, 95% CI=0.04-0.38] (p=0.000) were the most important associated factors of dental erosion. Conclusions: The prevalence of dental erosion in the study sample was 33.8% while dental erosion experience was associated with frequency and habits of consumption of some dietary components.

** Key words:**Prevalence, tooth erosion, risk factors, adolescents.

## Introduction

Tooth erosion (TE) has been defined as the physical result of a localized, chronic, pathologic and irreversible loss of dental hard tissue caused by acids or chelants without bacterial involvement (1).

TE is frequently encountered in dental practice, affected people of all ages and describes the condition of dental hard tissue. It may also be associated with dentine hypersensitivity, root caries, abrasion and gingival recession in case of the exposure of the root surface to the oral environment (2).

The aetiology of TE is multi-factorial and the result of more than one factor acting together. A wide range of factors has been identified as significantly associated with TE including chemical, biological and behavioural factors (3-5).These factors seem to influence the erosive process, making it difficult to identify the risk factors.

The most important factors that have been associated with TE are intrinsic and extrinsic, however interactions between individuals susceptibility factors, such as salivary characteristics and tooth/ tissue anatomy may play an important role in the development of erosive lesions (3,4,6).

The intrinsic factors include diseases that cause vomiting or regurgitation, such as gastro-oesophageal reflux, anorexia and bulimia nervosa, or illnesses that cause a reduction in saliva flow (3) while the extrinsic ones in-clude dietary habits (acidic drinks and foods) (3,7), occupational factors (occupation around acidic/chlorinated industrial environment) (3), sports (swimming in heavily chlorinated pools) (7), medicaments (acidic drugs or drugs that cause reduction in salivary flow) (4) and lifestyle habits (swallowing and drinking habits, consumption of acidic beverage at bedtime) (5,7). In addition, TE is influenced by educational, cultural and geographical factors (7).

A number of epidemiological studies have investigated the role of the mentioned factors in the development of erosive lesions in children and adolescents (6,8-10). However, similar investigations have not been carried out in Greece; therefore it is important to collect detailed information of this condition in order to assess the epidemiology, identify the aetiological factors and establish preventive measures. The present cross-sectional observational study was designed to assess the prevalence of TE and the associations among medical history, dietary habits, lifestyle factors and TE in a sample of adolescents in Greece.

## Material and Methods

Subject population

Study population consisted of 770 adolescents, 374 boys and 396 girls, 13 to 16 years of age. The sample were adolescents attending maintained schools who had reached the age of 13 to 16 years old but had not attained their 17th birthday on the date of examination.

The Greek Ministry of Health and the Greek Dental Association organize dental surveys for schoolchildren and adults annually, in order to assess the prevalence of diseases such as dental caries and periodontitis, the oral hygiene level and the treatment needs of the Greek population. All the participants complete an oral health questionnaire and undergo an oral clinical examination in several private practices without charge. This precondition is an important motivation in order to create a representative random study sample.

As part of the mentioned National Oral Health Survey the present study was carried out between November 2010 to May 2011. It is important to highlight that the topic of the present study was not included in the National Oral Health Survey. Therefore, the participants of the present study completed an additional questionnaire and underwent an oral clinical examination in a private practice.

Study population divided into two groups: erosion presence group (EPG) which showed erosion in at least one tooth surface and erosion absence group (EAG) which did not show any type of erosive lesion.

Selection Criteria

The selection criteria of the participants comprised adolescents who were not with orthodontic appliances, enamel defect accompanied by a loss of tooth substance, and fractured or missing teeth of the incisors or the first maxillary molars. These situations could lead to overor underestimate the prevalence of TE and the possible associations that are under consideration. The participants were in good general health as estimated by a general health questionnaire and confirmed by their parents/guardians.

Questionnaire

Before the clinical examination all adolescents filled in a selfadministered questionnaire regarding that aimed to establish which aetiological factors were associated with TE.

The questionnaire included variables such as age, gender, data regarding the general medical history of the sam-ple with reference to medication and chronic disorders, drink and food items which had erosive potential and the consumption of intake, which was classified as: low consumption (1-7 times per week) and high consumption (22 or more times per week).

Questions regarding chronic disorders included gastric disorders, diabetes mellitus, asthma, juvenile rheumatoid arthritis and medication included drugs for asthma treatment, aspirin (acetylsalicylic acid), vitamin C, and drugs that causes reduction in salivary flow rate (i.e. atropine hydrochloride, etc.)

Other questions asked whether acidic drinks were consumed at bedtime and if existed the habit of holding the drink in the mouth before swallowing and the habit of swimming in chlorinated pools. Regarding the habit of holding the drink in mouth, it was not possible to estimate the period (in seconds) that the dietary component (drink) was kept in mouth before swallowing. Consequently, the question was if they consume the examined drinks as they consume water (rinse with these before swallowing).

Clinical examination

The clinical examinations were performed in a private dental practice, using a conventional dental unit and illumination. One well-trained and calibrated dentist who was also registered as an active member in the Hellenic Society of Periodontology (HSP) and the European Federation of Periodontology (EFP) performed the examinations.

The clinical examination included the evaluation of the labial and palatal surfaces of the maxillary permanent incisors (# 11,12,21,22) and the occlusal surface of the first permanent molars (# 16,26,36,46) according to validated index proposed by the UK National Diet and Nutrition Survey (NDNS ) (11).

In order to assess the prevalence of TE, only enamel was involved and erosive lesions were considered with the following clinical characteristics: wide, shallow, U-shaped lesions with a smooth surface and no clear angles (1).

Initially the teeth and gingiva were dried with compressed air gently and the mentioned surfaces observed for erosive lesions carefully.

Ethical consideration

The present study was not an experimental one. In Greece only experimental studies must be reviewed and approved by authorized committees (Dental Schools, Greek Dental Association, Ministry of Health, etc.).

An informed consent letter regarding the aim and importance of the study was signed by the adolescents and the parents/guardians before starting the survey, which assured that children participated in the study on their own accord.

Reproducibility

A randomly chosen sample of 80 (10%) adolescents was reexamined clinically by the same dentist in order to establish the intra-examiner variance. After consideration of the code numbers of the double examined adolescents no differences were recorded between the 1st and the 2nd clinical assessment.

Statistical Analysis

The individual was the statistical unit in order to estimate the prevalence of TE.

Statistical analysis of questionnaire items was performed by using a multiple logistic regression analysis model to identify which variables were best associated with TE.

A stepwise selection procedure was used to investigate the influence of risk factors to the outcome of erosion. A two-step approach was used for this aim. First, bivariate analysis was used to test the relationship between TE and the associated factors. Thereby, the criterion for the independent variables to enter the model was set at 0.25. In addition, odds ratios with 95% confidence intervals (CI) were used to assess the bivariate relationships among the examined variables. Then, the mentioned model was used to analyse the factors that were independently related to the presence of TE. The variables after the bivariate analysis were entered into the model in a forward process and then in a back-ward fashion in order to find out which final variables could be considered as risk factors of TE.

The data analysis was performed using the statistical package of SPSS ver.17.0 (SPSS Inc, Chicago, IL, USA). A p value less than 5% (p<0.05) was considered to be statistically significant.

## Results

The total number of the adolescents who visited the private practice during the determined period for their an-nual dental follow-up was 834; however, 770 of them met the mentioned selection criteria giving a response rate 92.32%. The mean age of the sample of the study was 14.2 ± 0.4 years.

A sample of questions for the questionnaire relating medical history, dietary habits and lifestyle factors is shown in  Table 1.

**Table 1:**
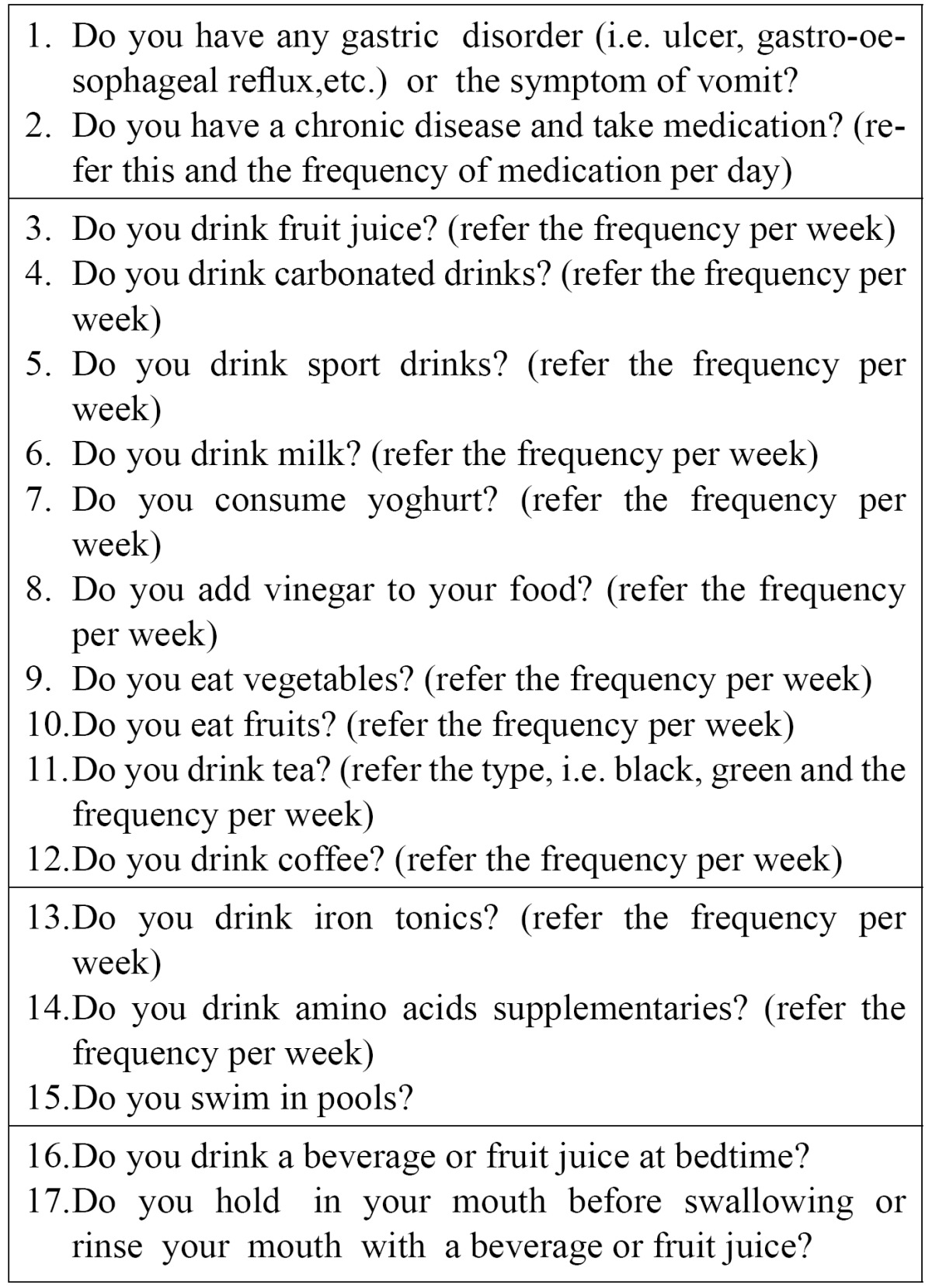
A sample of questions for the questionnaire relating medical history, dietary habits and lifestyle factors.

A total of 260 patients were diagnosed as having TE giving an overall prevalence of 33.8% (33.1 % in boys and 34.3% in girls, no statistically significant difference, p= 0.727), while the overall boys to girls ratio was 1:1.1 (p= 0.852) ( Table 2).

**Table 2:**
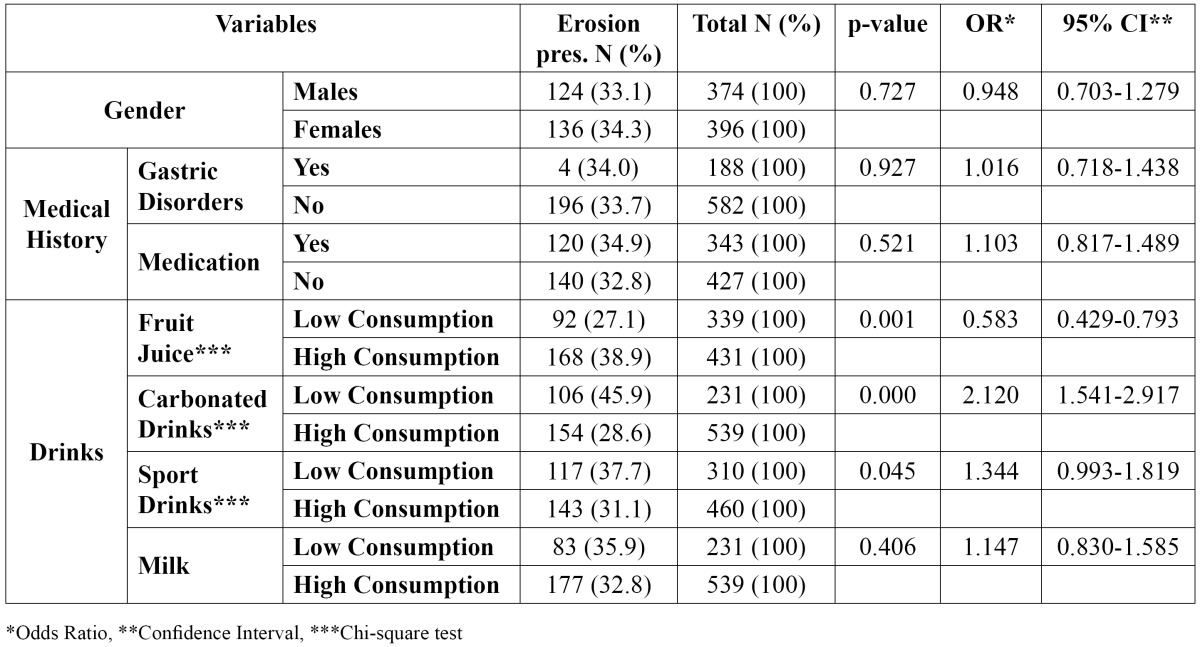
Association among dental erosion gender/medical history/drinks ingestion.

The EP group was consisted of 124 boys and 136 girls and the EA group was consisted of 250 boys and 260 girls. The results showed that factors such as gastric disorders (p= 0.927) and medication (p= 0.521) were not associated with TE according to the bivariate analysis model. ( Table 2).

The consumption of drinks and food was dichotomized in high (three per day; more than three per day) and low (less than once per day; never or rare) consumption.

Also, the consumption of other acidic components is shown in  Table 2 and  Table 3.

**Table 3:**
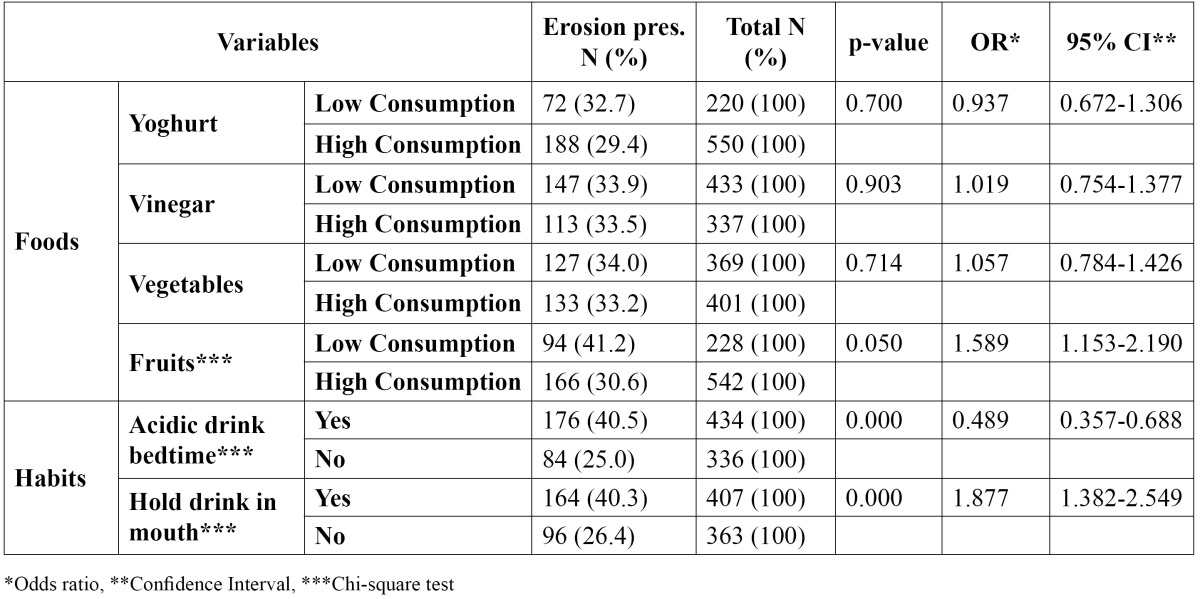
Association among dental erosion and foods consumption/drinking habits.

A significant association was recorded between the intake of fruit juice (p= 0.001), carbonated drinks (p= 0.000), sport drinks (p= 0.045), fruit consumption (p= 0.050) and the occurrence of TE ( Table 2, Table 3).

Consumption of an acidic drink at bedtime was reported by 40.5% of the adolescents. A statistically significant difference in the experience of erosion between those who consumed an acidic drink at bedtime and those who did not, was recorded (p=0.000). The habit of retaining the drink in mouth before swallowing was observed in 40.3% of the adolescents and it could be related to the occurrence of TE (p=0.000) ( Table 3). A group of questions such as consumption of green/black tea, coffee, iron tonics, amino-acids supplementaries and swimming in chlorinated pools were excluded from the statistical analysis because of the extremely low number of adolescents who consume the mentioned components or swim in pools.

The association between TE and the possible risk factors was analysed by the multiple logistic regression analy-sis model. The variables that entered the model (step one) were the following: consumption of fruit juice, carbo-nated and sport drinks, consumption of fruits and the habits of ingestion acidic drink at bedtime and holding drinks in mouth ( Table 2 and  Table 3). The final model (backward method) included only four variables: fruit juice (p= 0.000) and carbonated drinks consumption (p= 0.011), the habit of ingestion acidic drink at bedtime (p= 0.000) and the habit of holding drinks in mouth (p= 0.002) ( Table 4).

**Table 4:**
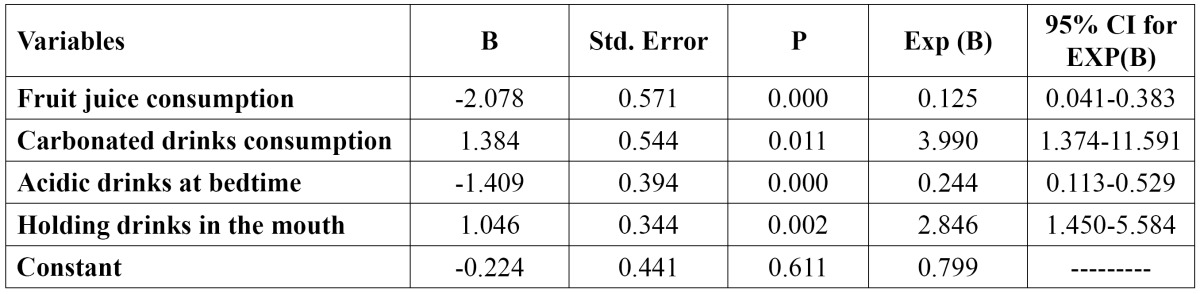
Final association between DE and several aspects (backward method).

## Discussion

The prevalence of TE in the current study was not similar to the results of other investigations. However, the prevalence ranging from 5.5 % to 100.0 % was determined during recent studies on children and adolescent samples in different countries (7,8,12-15).

The variation in prevalence among these studies may be explained by several factors. First, the different criteria used in the various studies could be at least partly the reason for this discrepancy. The tooth wear index (TWI) is the most extensively adopted index to measure TE, but it can overestimate the prevalence of this condition because it is not specific for TE and consequently measures different types of tooth wear. In addition, it is often difficult to distinguish the three main forms of tooth wear, i.e. erosion, attrition and abrasion (16)and it is likely that all three processes may have been included in some of the cases examined in the current study. Second, it is difficult to compare the results of prevalence studies when different teeth are included in the measurement method (17).

On the other hand the permanent dentition analysed in different investigations shows TE at ages ranging from 3 to 65+ years and this finding may also influence the results through differences in the time of exposure to risk factors. Standardization of the indices, ages and the teeth examined would facilitate such comparisons. Furthermore, socioeconomic, cultural and geographical factors could influence the outcome of prevalence data (17). In this investigation, no difference was observed in the prevalence of TE between boys and girls, finding that is in agreement with the findings of a previous investigation (13). However, other studies have recorded a significantly higher prevalence of exposed dentin in boys than in girls (8,14,15) and only in one study by Wang et al. (6) more girls than boys showed tooth erosion. The absence of significant difference in the prevalence of TE between boys and girls in the current study may be explained by similar patterns of exposure to risk factors in the study sample.

Gastric disorders and diseases such as anorexia nervosa and bulimia in which vomit is the main symptom are causative factors of TE according to previous studies (16,18), findings that were not in agreement with the re-sults of the current study. Only in one study (6) vomit as a symptom of gastric disease was not associated with TE.

According to the literature the use of chronic medication with an acidic composition such as aspirin (for use by active juvenile rheumatoid arthritis patients), vitamin C, drugs prescribed for the treatment of asthma or that causes reduction in salivary flow rate could increase the risk for TE (3,19,20).

The consumption of vitamin C has been considered a risk factor for TE, whereas previous investigations showed that the risk for TE is increased by the chronic consumption of vitamin C (9,21). No frequent use of vitamin C and other drugs was recorded in the current study and no significant differences were observed between the group with and without TE. Wang et al. (6) reported similar findings.

It is also important to highlight that few studies have been carried out regarding the influence of the mentioned medication on TE in children and adolescents.

The frequent consumption of carbonated drinks was shown to be strongly associated with TE, finding that was in agreement with those observed in previous studies (6,12,21,22). However, other studies (8,23) did not report such an association.

Mungia et al.(12) and Owens (24) reported that carbonated drinks including soda have strong erosive potential on teeth, as contain a lot of acid such as citric, phosphoric and carbonic acids which can rapidly dissolve enamel of teeth.

The modern lifestyle has changed the quality of dietary habits and has caused an increase in the consumption of carbonated beverages, fruit juices and sport drinks among children and adolescents. Especially, the consumption of carbonated drinks was increased substantially in the last years in all around the world mainly in children and adolescents because of low cost and high availability (11).

 Citric acid which is the main component of citrus fruits and its juices has higher potential to cause TE, than other types of acids, possibly because its chelating action on calcium enamel continues with the pH increases (11).The frequency of consumption of juice fruits was significantly associated with the occurrence of TE in the current study, finding that was in agreement with those reported in previous studies (9,18,21,22). Other investi-gations have recorded no significant associations between juice fruit consumption and TE (23,25).

The consumption of sport drinks has been increased mainly in adolescents who frequently take physical activities, because it rehydrates the organism and rapidly replace the mineral loss that is lost during transpiration. Owens (24) observed that these energy drinks had strong erosive potential on teeth possibly due to a high buffering capacity. The consumption of sport drinks was associated with the occurrence of TE in the current study and in previous studies (21,22), however, was not associated with it in other studies (6,9,25).

These discrepancies among the juice fruits and sport drinks consumption and TE could be attributed to the limitations mentioned above.

Milk has a protective effect against erosion, since it contains high concentration of calcium and phosphate in its composition and has a neutral pH (7.0) (26) . Similarly, Nahás Pires Corrêa et al. (10) observed that milk consumption was associated with a lower prevalence of TE and seemed to protect against this condition. Also, O’Sullivan and Curzon (9) observed that the low consumption of milk was considered a risk factor for TE.

In the present study TE was present in individuals with low and high consumption and this factor could not be considered as a protector factor against TE. In addition, El Aidi et al. (21) recorded no difference in the consumption of milk between the groups with and without TE.

On the other hand yoghurt or another milk-based food may have an erosive potential, in case it has a low content of calcium and/or phosphate and a low pH (11,27). In the current study no associations were recorded regarding yoghurt and milk-products consumption and TE.

Vinegar consumption has a high potential to cause TE as has low pH, according to several studies (9,21,26). However, no association was observed between vinegar consumption and TE in the current study.

The consumption of fruits was related to TE in the present study. Similarly, some authors observed that citric fruits are dietary components of high impact in the occurrence of erosive lesions (9,10,18), whereas other authors have found no associations between fruit consumption and TE (6,23).

The results of the current study showed no significant difference in TE prevalence between vegetarian and non-vegetarian adolescents which was in agreement with the study by Al-Dlaigan et al.(22). However, El Aidi et al. (21) recorded an association between vegetable consumption and occurrence of TE. The mentioned discrepancy shows that further research is necessary on this issue.

In the literature, many studies have shown that individuals with the habit of holding drinks in the mouth especially, carbonated beverages, before swallowing could have a greater susceptibility to TE (6,9,11,27).

These observations were in agreement with the results of the present study. Holding drinks in the mouth before swallowing causes a marked pH drop at the tooth surface and increases the risk of erosion (28).

Only in a study by Wang et al. (6) no association between the mentioned habit and TE was recorded.

Another condition that could predispose to TE is the ingestion of acidic drinks at bedtime. Zero et al. (5) reported that exposure to erosive agents at night is particularly destructive because of the nocturnal absence of salivary flow and Wang et al. (6) confirmed that the salivary flow is diminished during this period.

In the current study the consumption of acidic drinks at bedtime was associated with TE, finding that was in agreement with the findings reported in a study by Moazzez et al. (27), whereas Milosevic et al. (25) and Wang et al. (6) did not observe such an association. It is clear that there is a need for more definitive research in this issue.

As mentioned, some possible risk factors such as consumption of tea, coffee, iron tonics, amino acids supplementaries and swimming in chlorinated pools were excluded from the statistical analysis as the number of adolescents which consume those components or swim in pools was extremely low.

However, previous investigations have recorded that the mentioned factors were, in many cases, associated with the occurrence of TE (21,27,29-30).

TE is a multi-factorial condition and there are many factors that were not investigated in the present study and could be associated with TE, such as the protective effect of saliva and the association between TE and abra-sion/attrition. It could be assumed also that other factors such as cultural, social, occupational and interand intra-individual host factors might be relevant in the occurrence of TE.

The identification of aetiological factors associated with TE is very important for the establishment of preventive measures. Epidemiological and case-control studies have been developed in the last years to elucidate possible causal determinants for TE.

These studies could show associations and indicative risk factors but they could not identify the aetiological factors, because for this a prospective study could be necessary. Other studies are still necessary to explain the aetiology of TE, focusing in the biological, chemical and behavioural factors involved in order to implement adequate preventive policies.

In conclusion, the present investigation provided evidence that TE is a significant problem in Greek adolescents. TE should receive more attention that promotes awareness in dentists to make an early diagnosis and to identify the different aetiological factors of this condition. Adolescents with high consumption of fruit juices and carbonated drinks and had the habits to consume acidic drinks at bedtime and holding these drinks in the mouth before swallowing tended to have more TE.

A strategy of offering preventive care, including more campaigns promoting a healthier lifestyle for those at risk of TE and a regular dental follow-up, should be conducted for school-children and adolescents in order to eliminate the medical burden of the government and families.
